# Neuromuscular changes during a four-week individualized in-season strength-training program based on the dynamic strength index in youth basketball players: A quasi-experimental study

**DOI:** 10.1371/journal.pone.0350739

**Published:** 2026-06-15

**Authors:** Casado-Martínez P, Romero-Moraleda B, González-García J

**Affiliations:** 1 Faculty of Health Sciences, Universidad Francisco de Vitoria, Pozuelo de Alarcón (Madrid), Spain; 2 Faculty of Health Sciences, Universidad Camilo José Cela, Madrid, Spain; 3 Sports Performance Area, Royal Spanish Football Federation, Madrid, Spain; Ordu University, TÜRKIYE

## Abstract

The dynamic strength index (DSI) is commonly used as a neuromuscular profiling metric to inform individualized strength-training decisions; however, evidence describing short-term in-season changes in DSI and constituent neuromuscular variables in youth team-sport athletes remains limited. This quasi-experimental applied study quantified neuromuscular changes during a four-week individualized in-season strength-training program in 21 youth basketball players (age: 16.4 ± 1.3 years). Players were stratified into high (>0.57), neutral (0.45–0.57), or low (<0.45) baseline DSI tertiles to prioritize training emphasis (maximal strength, mixed, or ballistic/plyometric, respectively). Participants completed three individualized sessions per week alongside regular basketball training. Pre- and post-intervention measures included countermovement jump (CMJ) height and peak force, isometric squat (ISOBelt) peak force, and estimated one-repetition maximum (1RM) back squat. DSI was subsequently calculated from CMJ and ISOBelt peak force values. The high DSI tertile showed increases in ISOBelt peak force (*p* = 0.004, *d* = 2.10) and estimated 1RM back squat (*p* = 0.002, *d* = 1.84), accompanied by a reduction in DSI (*p* < 0.001, *d* = −1.56), whereas CMJ outcomes showed no statistically significant changes across tertiles (all *p* > 0.05). The neutral tertile showed an increase in estimated 1RM back squat (*p* = 0.002, *d* = 0.89), although no significant interaction effects were observed. During the competitive season, DSI-based profiling was associated with distinct within-tercile neuromuscular changes over four weeks; findings should be interpreted as observational outcomes within an applied in-season context and may assist practitioners in guiding individualized in-season strength-training prioritization.

## Introduction

In team sports, the development of strength and power is a key objective to enhance performance-related physical capacities. This is particularly relevant in basketball, where actions such as jumping, sprinting, accelerating, decelerating, and changing direction are key determinants of performance [1]

Traditionally, resistance training programs have followed generalized approaches that do not account for inter-individual variability in neuromuscular characteristics. However, training individualization is often advocated in applied settings to better match training stimuli to athlete characteristics, although the magnitude of benefit may depend on baseline characteristics, training status, and concurrent training load [2]. Within this framework, the DSI has been proposed as a useful metric to evaluate an athlete’s force-production profile. The DSI is calculated as the ratio between peak force achieved during ballistic tasks (e.g., vertical jump) and peak force obtained during maximal isometric actions [3]. Training recommendations based on DSI have been proposed in the literature; however, commonly cited cut-offs (e.g., low vs high DSI) are highly dependent on the specific testing protocols and athlete population. Importantly, DSI values should not be interpreted in isolation, as similar ratios may arise from markedly different levels of maximal and ballistic force. Therefore, practitioners are encouraged to interpret DSI alongside its constituent variables to guide training prioritization [4].

DSI calculation requires two primary variables: peak force from a ballistic task and peak force during a maximal isometric task. While the isometric mid-thigh pull (IMTP) remains the most widely used method for assessing maximal isometric force, alternative approaches have been proposed. The isometric squat using a belt-based system (ISOBelt) has recently emerged as a practical, reliable, and sensitive alternative, offering enhanced lower-limb specificity, greater force output, and improved comfort—particularly in younger or less experienced athletes [5]. Recent evidence by Tsopanidou [6] suggests that the ISOBelt configuration produces higher peak forces and is better tolerated than the IMTP, without compromising the reliability or validity of the DSI. Despite both assessments targeting isometric strength, they differ biomechanically, which can substantially impact the resulting DSI score. In fact, peak forces measured during ISOBelt have been shown to exceed IMTP values by 9–28% requiring test-specific interpretation when establishing DSI thresholds [7].

Although short-term resistance training interventions (5–12 weeks) using strength or ballistic-oriented protocols have been shown to improve performance in youth athletes, these programs are typically non-individualized [8,9]. More recently, individualized training approaches informed by DSI have been proposed as a framework for aligning training emphasis with an athlete’s neuromuscular profile, with some studies reporting favorable performance-related outcomes [2,10]. For instance, [2] reported that basketball players who followed a DSI-based training program achieved superior improvements in sprinting, jumping, and change-of-direction performance compared to those in a control group following a generic training plan. These findings support the potential utility of DSI-informed strategies for optimizing training prescription.

Despite these findings, it remains unclear whether DSI itself is sufficiently sensitive to detect short-term neuromuscular changes during in-season training, particularly in youth team-sport athletes under applied conditions. To our knowledge, no previous study has examined short-term in-season changes in DSI alongside its constituent neuromuscular variables within an individualized training framework in youth basketball players. The purpose of this study was to quantify changes in DSI, countermovement jump (CMJ) height and peak force, isometric squat (ISOBelt) peak force, and estimated one-repetition maximum (1RM) back squat following a four-week individualized in-season strength-training program based on baseline DSI tertiles in youth basketball players.

Based on the principle of velocity specificity, it was hypothesized that athletes classified within the high DSI group would exhibit greater increases in maximal strength-related variables, whereas those classified within the low DSI group would demonstrate comparatively greater changes in CMJ-related outcomes, acknowledging the applied and non-controlled nature of the study.

## Methods

### Experimental approach to the problem

To address the study purpose, a quasi-experimental, longitudinal pre-post design without a control group was implemented. This design was selected to reflect an applied in-season training context, where the inclusion of a non-training control group was not feasible because participants belonged to a competitive team and continued their regular in-season training and competition schedule. Withholding training from a subgroup of athletes during the competitive season was not considered practical or ethically appropriate in this applied setting. Participants were stratified into three DSI tertiles based on baseline values to prioritize training emphasis over a four-week period. All participants completed two familiarization sessions and two testing sessions (pre and post) to monitor changes in the primary outcomes.

### Participants

The sample size was determined using G*Power 3.1.9.7 (Heinrich Heine University, Düsseldorf, Germany) for a repeated-measures ANOVA (within-between design), assuming an effect size of *f* = 0.30 (η² ≈ 0.08), based on conventional thresholds for repeated-measures designs, an alpha level of 0.05, and a statistical power of 80%. The analysis indicated that a minimum sample size of 21 participants was required. Consequently, 21 young basketball players, 12 males and 9 females, were recruited (mean age: 16.42 ± 1.34 years; height: 176.22 ± 8.04 cm; weight: 69.48 ± 12.51 kg; 1RM squat: 106.58 ± 16.28 kg).

Participants were classified as trained youth athletes, with regular participation in competitive basketball and at least one year of structured resistance training experience. Those with injuries within the four weeks preceding the study or who sustained injuries or illnesses that prevented them from completing all sessions were excluded. The study protocol was approved by the Ethics Committee of Universidad Camilo José Cela (CEI-UCJC, Madrid, Spain; approval code: 2021/02) and was conducted in accordance with the Declaration of Helsinki. Written informed consent was obtained from all participants and from their parents or legal guardians prior to participation.

### Procedures

#### Familiarization sessions.

Two familiarization sessions were conducted one week prior to the intervention. The warm-up protocol was based on the RAMP method (Raise, Activate, Mobilize, Potentiate) [11] and included general locomotor activities, mobility drills (ankles, knees, hips, shoulders), two sets of bodyweight exercises (squats, glute bridges, lunges, push-ups, lateral lunges, and single-leg Romanian deadlifts), and a potentiation phase comprising bilateral and unilateral pogos, five CMJs, and five rebound-style CMJs.

In the first session, estimated 1RM in the back squat was derived using the load–velocity profiling method with three submaximal loads (60%, 70%, and 80%) based on each participant’s self-estimated 1RM [12]. Additionally, participants performed three CMJs and two 5-second ISOBelt trials with the highest value retained for analysis.

#### Testing Sessions.

Pre- and post-intervention testing followed an identical protocol, beginning with the same standardized warm-up described above. After a three-minute rest period, participants performed three CMJs and one 5-second ISOBelt trial. A single trial was retained during testing to reduce fatigue and minimize interference with the subsequent training schedule in this applied in-season setting. The DSI was calculated as the ratio of CMJ propulsive peak force to isometric squat peak force.

**Fig 1:**
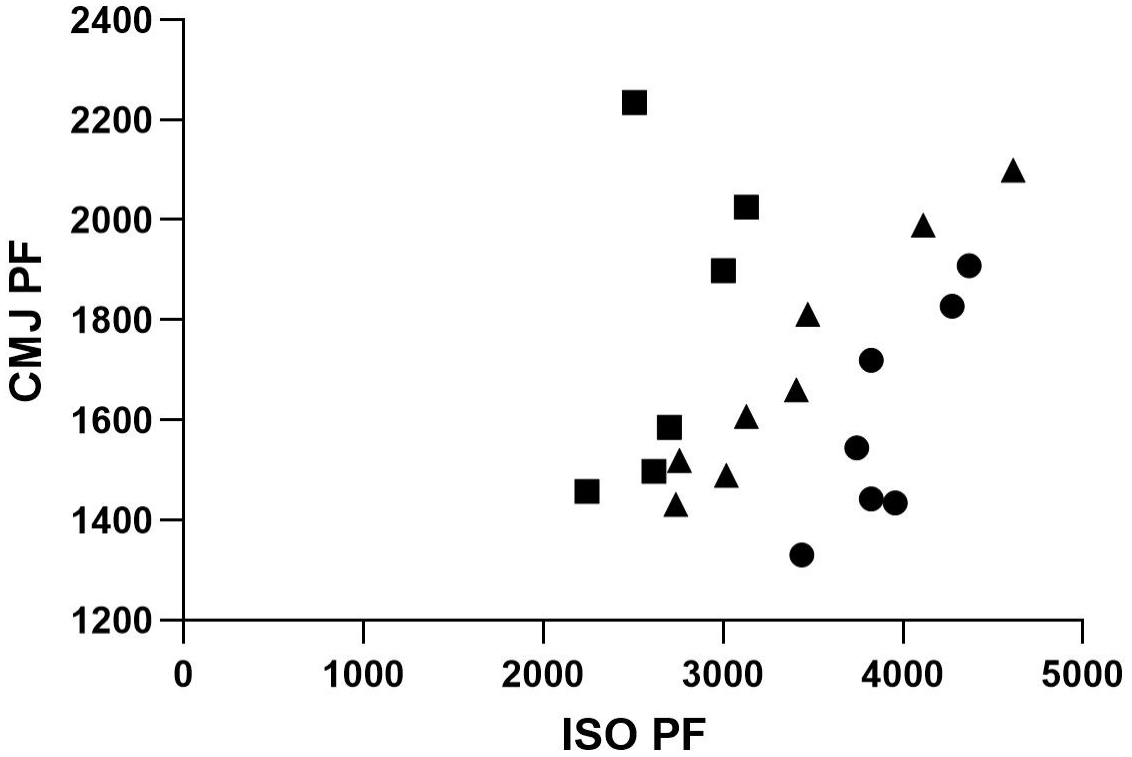
Individual distribution of isometric peak force (ISO PF) and countermovement jump peak force (CMJ PF) values for the Dynamic Strength Index (DSI) tertile classification.

Participants were stratified into low (<0.45), neutral (0.45–0.57), and high (>0.57) DSI groups using baseline sample-specific tertiles. We adopted sample tertiles rather than universal thresholds because our isometric test was the ISOBelt at 130° knee flexion, which typically yields higher peak forces than the IMTP (≈9–28%), thereby shifting DSI values downward relative to IMTP-based norms [7]. This approach ensured context-specific interpretation and more accurate group allocation for individualized prescription (Fig 1).

All testing sessions were conducted at the same time of day for each participant to minimize circadian variability. In addition, athletes were instructed to maintain consistent sleep and dietary habits throughout the intervention period and to refrain from any strenuous physical activity outside the prescribed basketball and resistance training sessions.

All assessments were conducted using ForceDecks FD4000 dual force platforms (ForceDecks, London, UK), sampling at 1000 Hz. Data were processed using ForceDecks software. Force plates were zeroed prior to each testing session. For CMJ, participants kept their hands on their hips throughout the jump, avoided knee flexion in flight, and landed with minimal heel contact. Outcome variables included jump height (from impulse-momentum method) and peak force [13]. Verbal instructions and standardized encouragement were provided during all trials. Trials were repeated if technical execution criteria were not met.

ISOBelt was performed on a custom device developed for this study. Athletes stood on force plates, attached by a chain to a fixed structure via a waist-worn belt, positioned at the iliac crest. A knee angle of 130° was established using a goniometer, following Brady et al. [7]. Participants were instructed to exert maximal force as quickly and forcefully as possible while maintaining a fixed body position. Peak force during the 5-second trial was recorded.

### Training intervention

Over the four-week period, participants completed three resistance training sessions per week in addition to routine basketball practices (four per week) and one official game per weekend. The intervention was conducted during the competitive in-season period, while participants maintained their regular weekly schedule of team practices and official matches. In collaboration with team coaches, on-court basketball training load (i.e., session frequency, duration, and content) was pre-planned and kept consistent across the four-week intervention period to minimize potential confounding effects of external load on neuromuscular responses. Operationally, the high-DSI group followed a maximal-strength emphasis (higher relative loads), the low-DSI group prioritized ballistic/plyometric and lighter-load power-oriented work, and the neutral-DSI group combined both emphases, as detailed in Tables 1,2. All resistance training sessions lasted approximately 60 minutes (including warm-up). Rest intervals were standardized to 2–4 minutes between sets. Prescribed loads were adjusted weekly based on the planned %RM progression and coach supervision to ensure target intensity was achieved. All sessions were supervised by certified strength and conditioning coaches to ensure correct technique and adherence to the prescribed intensity.

**Table 1:** Weekly training content for each Dynamic Strength Index (DSI) group across the four-week intervention period.

HIGH DSI (DSI > 0.57)				
Training 1	Week 1	Week 2	Week 3	Week 4
Split squat	3x6 70%RM	3x6 75%RM	3x5 77%RM	3x4 80%RM
Hip thrust	3x5 75%RM	3x4 80%RM	3x3 85%RM	3x2 90%RM
Unilateral deadlift	3x6 70%RM	3x6 75%RM	3x5 77%RM	3x4 80%RM
**Training 2**	**Week 1**	**Week 2**	**Week 3**	**Week 4**
Squat	3x4 80%RM	3x3 85%RM	3x2 90%RM	3x2 92%RM
Deadlift	3x4 80%RM	3x3 85%RM	3x2 90%RM	3x2 92%RM
Hang power clean	3x4 80%RM	3x3 85%RM	3x2 90%RM	3x2 92%RM
**Training 3**	**Week 1**	**Week 2**	**Week 3**	**Week 4**
Maximal isometric squat	2x3x3“”	2x3x3“”	2x3x3“”	2x3x3“”
Squat	2x4 80%RM	2x3 85%RM	2x2 90%RM	2x2 92%RM
Hang power clean	2x3 80%RM	2x3 85%RM	2x2 90%RM	2x2 92%RM
**NEUTRAL DSI (DSI 0.45-0.57)**				
**Training 1**	**Week 1**	**Week 2**	**Week 3**	**Week 4**
Split squat	3x8 65%RM	3x8 70%RM	3x6 75%RM	3x6 77%RM
Hip thrust	3x6 75%RM	3x5 80%RM	3x4 85%RM	3x3 90%RM
Box jumps	3x6	3x8	4x6	4x8
**Training 2**	**Week 1**	**Week 2**	**Week 3**	**Week 4**
Squat	3x5 80%RM	3x4 85%RM	3x3 90%RM	3x2 92%RM
Deadlift	3x5 80%RM	3x4 85%RM	3x3 90%RM	3x2 92%RM
Horizontal jumps	3x6	3x8	4x6	4x8
**Training 3**	**Week 1**	**Week 2**	**Week 3**	**Week 4**
Hexa bar jump	3x5 20%RM	3x5 25% RM	3x4 30%RM	3x4 35%RM
Squat	2x6 60%RM	2x6 65%RM	2x5 67%RM	2x5 70%RM
Hang power jump	2x3 80%RM	2x3 85%RM	2x2 90%RM	2x2 92%RM
**LOW DSI (DSI<0.45)**				
**Training 1**	**Week 1**	**Week 2**	**Week 3**	**Week 4**
Split jumps	4x6 20%RM	4x6 25%RM	4x5 30%RM	4x5 35%RM
Hip thrust	4x6 60%RM	4x6 65%RM	4x5 67%RM	4x5 70%RM
Box jumps	5x6	5x8	6x6	6x8
**Training 2**	**Week 1**	**Week 2**	**Week 3**	**Week 4**
Drop jumps	4x6	4x8	5x6	5x8
Deadlift	4x6 60%RM	4x6 65%RM	4x5 67%RM	4x5 70%RM
Horizontal jumps	5x6	5x8	5x6	5x8
**Training 3**	**Week 1**	**Week 2**	**Week 3**	**Week 4**
Hexa bar jump	4x6 20%RM	4x6 25%RM	4x5 30%RM	4x5 35%RM
Squat	4x6 60%RM	4x6 65%RM	4x5 67%RM	4x5 70%RM
Pogo jumps	4x8	4x10	4x8	4x10

**Table 2:** Total training volume expressed in arbitrary units (AU) for each DSI group across the four-week intervention period.

HIGH DSI (DSI > 0.57)				
Training load (exercises* sets*repetitions*%RM = AU)				
Training	Week 1	Week 2	Week 3	Week 4
1	3645	3660	3075	2460
2	2880	2295	1620	1656
3	1120	1020	720	736
**Total**	**7645**	**6975**	**5415**	**4852**
	**Grand total (AU): 24887**			
**NEUTRAL DSI (DSI 0.45–0.57)**				
**Training**	**Week 1**	**Week 2**	**Week 3**	**Week 4**
1	2928	2904	2394	2228
2	2418	2574	1644	1136
3	1440	1665	1390	1593
**Total**	**6786**	**7143**	**5428**	**4957**
	**Grand total (AU): 24314**			
**LOW DSI (DSI < 0.45)**				
Training load (exercises* sets*repetitions*%RM = AU)				
**Training**	**Week 1**	**Week 2**	**Week 3**	**Week 4**
1	1950	2200	2244	2428
2	1494	1632	1668	1760
3	1952	2200	2240	2420
**Total**	**5396**	**6032**	**6152**	**6608**
	**Grand total (AU): 24188**			

**Note.** AU = exercises × sets × repetitions × relative load (%RM).

### Statistical analysis

Statistical analyses were performed using jamovi (Version 2.3.11; The jamovi Project, Sydney, Australia). Data were analysed using two-way repeated-measures analysis of variance (ANOVA), with time (pre, post) as the within-subject factor and DSI tertile (high, neutral, low) as the between-subject factor. Normality of residuals was assessed using the Shapiro–Wilk test. Homogeneity of variances for the between-subject factor was assessed using Levene’s test. Given that the within-subject factor included only two levels, sphericity was not applicable. Partial eta-squared (η²p) was reported as a measure of effect size and interpreted as small (0.01), medium (0.06), and large (0.14). Where significant time × group interactions were observed, post hoc comparisons were conducted to examine pre–post changes within each tertile, applying Bonferroni correction for multiple comparisons.

Within-tercile effect sizes for pre–post changes were calculated as Cohen’s d using the pre-test standard deviation for standardization (d = [post − pre] / SDpre) and are reported with 90% confidence intervals. Test–retest reliability was assessed using intraclass correlation coefficients (ICC) and coefficient of variation (CV), with interpretation based on established thresholds. Given the absence of a control group, findings were interpreted as observational monitoring outcomes.

## Results

Test–retest reliability values for the primary outcome variables are presented in Table 3. Intraclass correlation coefficients (ICC ≥ 0.96) and coefficients of variation (CV ≤ 8.44%) are reported.

**Table 3:** Reliability of the primary outcome measures.

Outcomes	Trial 1		Trial 2		SWC	ICC (95%CI)				CV (95%CI)				
	Mean	SD	Mean	SD		ICC	LL	UL	SEM	MDC 95%CI	MDC 90%CI	CV (%)	LL	UL
CMJ Peak Force	1703.24	330.32	1690.71	256.41	111.59	0.96	0.9	0.98	90.71	251.44	211.03	3.04	1.16	4.92
CMJ Height	31.29	6.83	31.24	6.31	1.27	0.97	0.92	0.99	0.82	2.26	1.9	2.08	0.79	3.36
ISOBelt Peak Force	3109.29	812.13	3374.43	670.72	522.59	0.97	0.92	0.99	306.67	850.05	713.43	8.44	3.21	13.68

For DSI, a significant main effect of time (F = 21.8, *p* < 0.001, η²ₚ = 0.548) and a significant time × group interaction (F = 13.2, *p* < 0.001, η²ₚ = 0.594) were observed. The main effect of group for DSI was not considered for interpretation, as groups were defined based on baseline DSI values. For isometric peak force, a significant main effect of time (F = 15.38, *p* < 0.001, η²ₚ = 0.461) and a significant time × group interaction (F = 5.41, *p* = 0.014, η²ₚ = 0.375) were observed, whereas the main effect of group was not statistically significant (F = 2.97, *p* = 0.077). For estimated 1RM squat, a significant main effect of time was observed (F = 49.26, *p* < 0.001, η²ₚ = 0.179), whereas neither the time × group interaction (F = 1.49, *p* = 0.252) nor the main effect of group (F = 1.32, *p* = 0.293) reached statistical significance. No significant main effects or interaction effects were observed for CMJ peak force or jump height (all *p* > 0.05).

**Fig 2:**
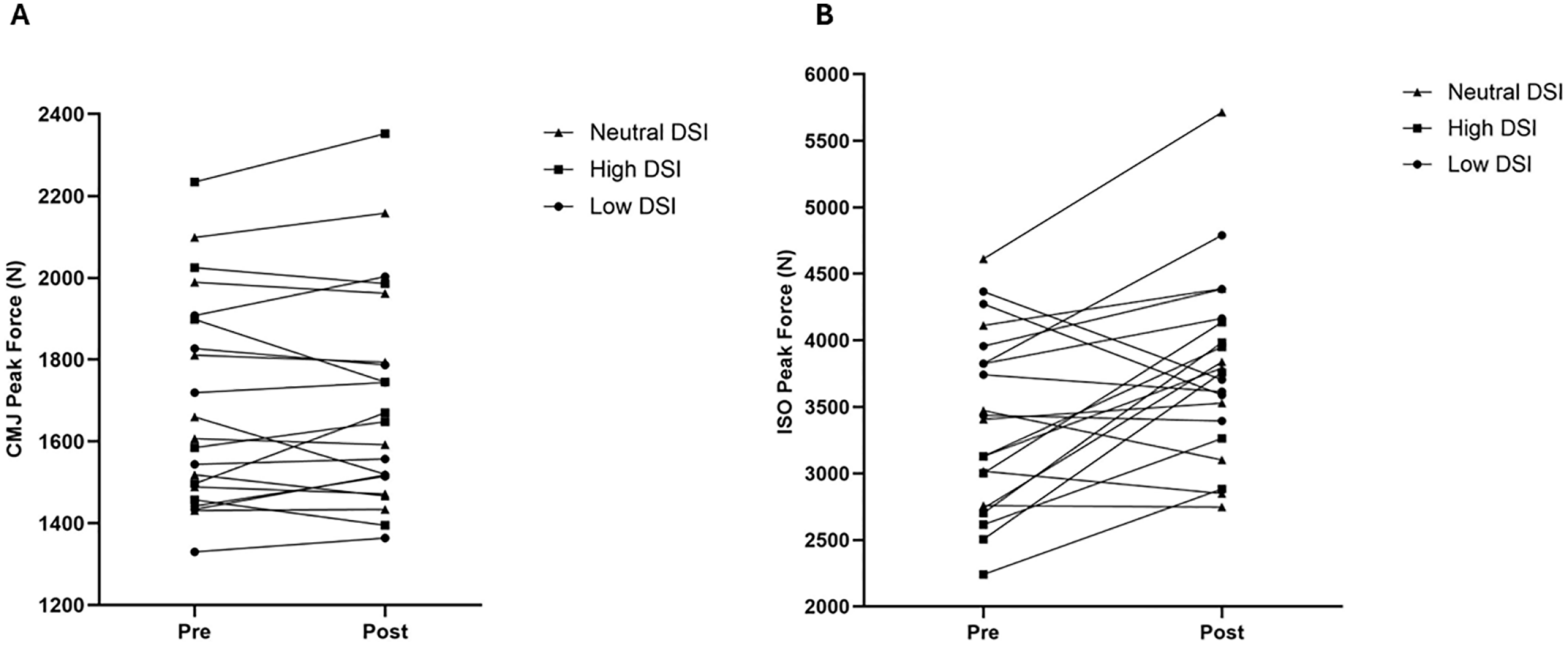
Individual pre-post changes in countermovement jump peak force (CMJ Peak Force) and isometric peak force (ISO Peak Force) according to DSI tertile classification (high, neutral, and low).

**Fig 3:**
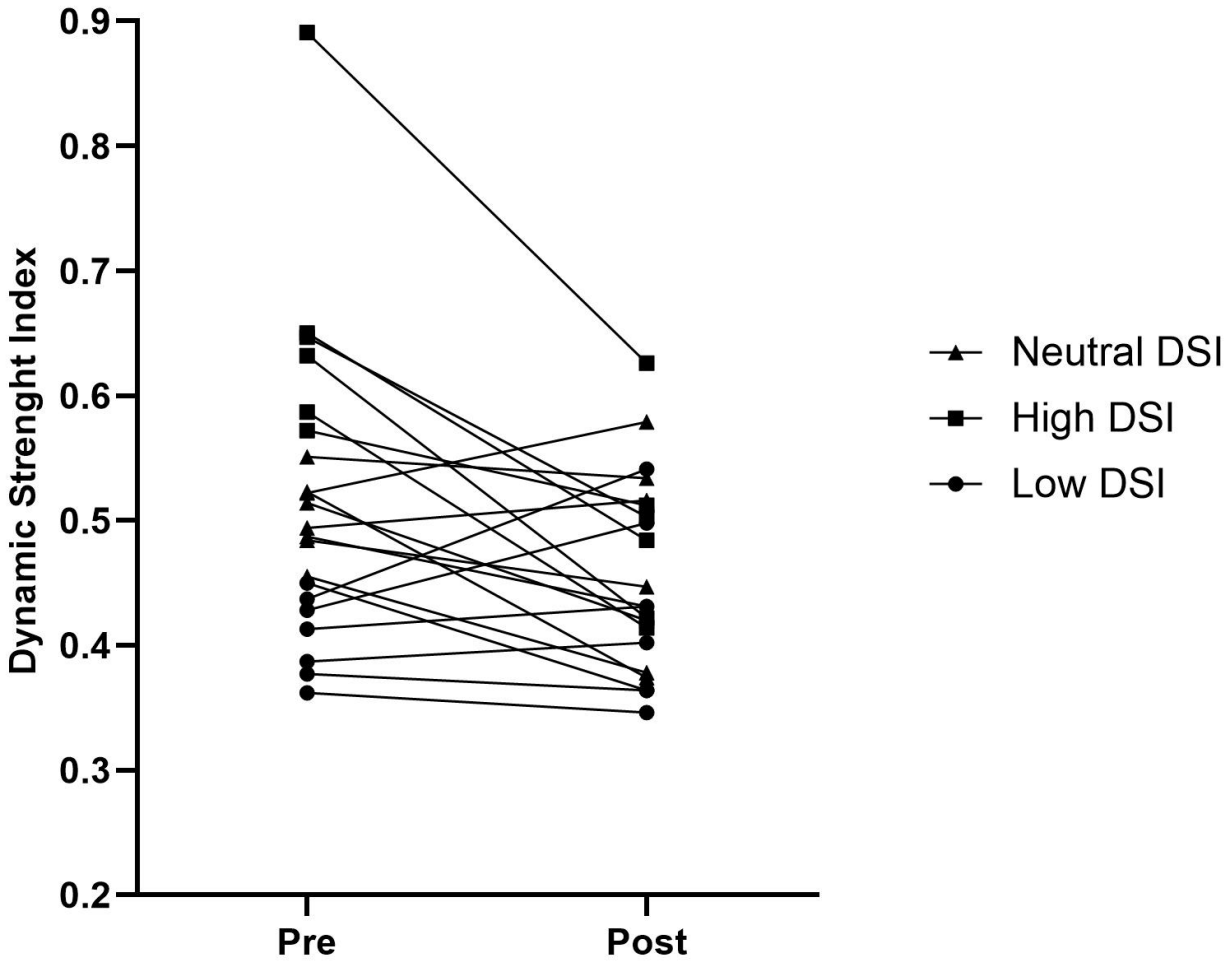
Individual pre-post changes in the Dynamic Strength Index (DSI) according to baseline DSI tertile classification (high, neutral, and low).

**Fig 4:**
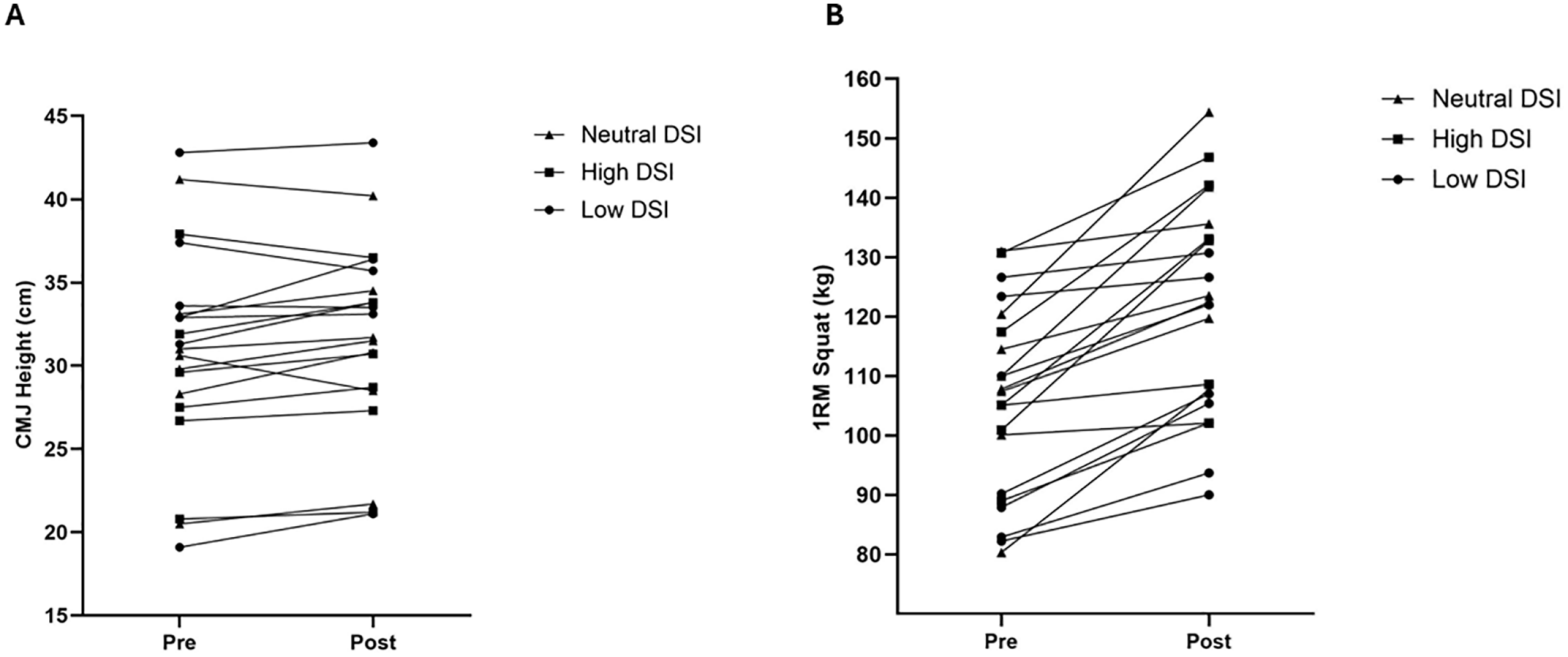
Individual pre-post changes in estimated 1RM back squat and countermovement jump height (CMJ Height) according to DSI tertile classification (high, neutral, and low).

Post hoc comparisons indicated expected between-group differences in DSI values, reflecting baseline-based group classification. A significant pre–post reduction in DSI was observed in the high DSI group (*p* < 0.001, *d* = −1.56, 90% CI [−2.01 to −1.11]). Individual pre–post changes in DSI across tertiles are presented in Fig 3. A significant pre–post increase in isometric peak force was observed in the high DSI group (*p* = 0.004, *d* = 2.10, 90% CI [1.65–2.56]). Individual pre–post changes in CMJ peak force and isometric peak force across DSI tertiles are shown in Fig 2. Although a significant main effect of time was observed for estimated 1RM squat, no significant time × group interaction was found. Descriptive within-group changes indicated increases in estimated 1RM in the high DSI group (*p* = 0.002, *d* = 1.84, 90% CI [0.45 to 3.18]) and the neutral group (*p* = 0.002, *d* = 0.89, 90% CI [0.45 to 1.33]); however, these changes should not be interpreted as differential responses between groups. Individual pre–post changes in estimated 1RM squat and CMJ height across tertiles are shown in Fig 4.

## Discussion

The primary findings of this study indicate that, over the four-week in-season period, players classified within the high DSI tertile exhibited increases in maximal isometric force and estimated 1RM back squat, accompanied by a reduction in DSI, whereas CMJ variables remained largely unchanged. These findings should be interpreted as observational changes within an applied training context rather than definitive effects of the intervention, whereas the neutral and low DSI groups showed no meaningful changes in the primary outcomes.

Consistent with our findings, Comfort et al. [10] and Pleša et al. [2] also reported reductions in DSI coinciding with increases in maximal isometric force production, which may reduce the ratio between ballistic and isometric force. Importantly, DSI values should not be interpreted in isolation, as similar ratios may arise from different combinations of maximal and ballistic force. Similarly, in agreement with both studies, no improvements were observed in the ballistic group for any CMJ variable. The absence of significant changes in CMJ performance may be explained by the short duration of the intervention and the limited exposure to ballistic-specific stimuli. During short-term interventions, neural mechanisms predominate, enhancing maximal force capacity prerequisite for power development, particularly in younger or less-experienced athletes, where initial strength gains may facilitate subsequent improvements in explosive performance [14,15]. In youth athletes, improvements in explosive performance typically require longer training durations or greater exposure to velocity-specific stimuli. Recent evidence in youth athletes indicates that improvements in jump performance generally emerge after 6–8 weeks of speed- or plyometric-oriented training [16,17].

A notable difference with previous literature is that CMJ peak force values in our sample were substantially lower than those reported by Pleša et al. [2], despite both studies involving basketball players. This discrepancy is likely attributable to the younger age and lower training experience of our participants (16.4 vs. 19.4 years), factors known to affect fast force production [18]. Another methodological difference is the use of the ISOBelt rather than the IMTP. Our approach yields 9–28% higher peak force values than the IMTP due to greater recruitment of hip and knee extensors and reduced contribution of the upper-body musculature. In our study, isometric forces (~3000 N) were markedly higher than those reported in IMTP-based protocols [2,10]. Consequently, the DSI cut-off values were lower (0.57 vs. ~ 0.80), highlighting how test selection directly influences interpretation of the index.

Although group means for CMJ variables did not change, the individual-response profiles (Table 4) indicate that several athletes in the high-DSI group—who followed a maximal-strength emphasis without ballistic-specific work—exhibited positive changes in jump performance. However, these individual changes were not sufficient to influence group-level outcomes. The presence of positive responders despite the absence of ballistic training is consistent with evidence that heavy resistance training (≥85% 1RM) can yield jump gains comparable to, or greater than, plyometric interventions [19]. Collectively, this pattern is consistent with theoretical models proposing maximal strength as an important determinant and foundation for subsequent power expression, particularly in youth athletes. However, given the applied design and short timeframe, these observations should be interpreted cautiously and primarily as monitoring outcomes rather than definitive training effects [14].

**Table 4:** Descriptive statistics, adjusted p-values, Cohen’s d, and individual responses for the primary outcome measures according to intervention subgroups.

Outcomes	Group	Pre-intervention	Post-intervention	*p*-value	Cohen’s d	Individual response (+/-/n)
**Mean ± SD**	**Mean ± SD**
CMJ Peak Force	High	1783.0 ± 317.0	1799.0 ± 331.0	1.000	0.04	2/1/3
	Neutral	1701.0 ± 243.0	1675.0 ± 268.0	1.000	−0.11	0/1/7
	Low	1601.0 ± 220.0	1641.0 ± 215.0	1.000	0.16	1/0/6
CMJ Height	High	29.1 ± 5.7	29.7 ± 5.35	1.000	0.10	3/1/2
	Neutral	31.5 ± 6.26	30.4 ± 5.72	1.000	−0.15	4/3/1
	Low	32.9 ± 7.21	33.9 ± 6.64	1.000	0.12	2/1/4
	High	2699.0 ± 325.0	3662.0 ± 488.0	**0.004**	2.10	6/0/0
ISO Peak Force	Neutral	3406.0 ± 660.0	3745.0 ± 968.0	1.000	0.40	3/1/4
	Low	3918.0 ± 319.0	3949.0 ± 509.0	1.000	0.04	3/2/2
	High	0.663 ± 0.116	0.493 ± 0.0769	**< 0.001**	−1.56	6/0/0
DSI	Neutral	0.504 ± 0.0296	0.460 ± 0.0752	1.000	−0.63	0/3/5
	Low	0.408 ± 0.0327	0.421 ± 0.0793	1.000	0.24	2/1/4

Adjusted p-values are reported; values of 1.000 reflect Bonferroni-adjusted results.

For the neutral and low DSI groups, the absence of improvements in jump metrics despite training is likely related to the limited duration and volume of the ballistic stimulus. Guimarães et al. [20] reported that four weeks of plyometric training in youth athletes did not result in significant increases in countermovement jump height. Similarly, Sozbir [21] observed increased neuromuscular activation after six weeks of plyometric training, but no significant gains in jump height were detected. In contrast, Gepfert et al. [22] found significant improvements in CMJ following eight weeks of plyometric training in young athletes, and Cherni et al. [23] showed that both loaded and unloaded plyometric training combined with change-of-direction sprints over eight weeks produced substantial increases in neuromuscular performance, including CMJ, in elite U-18 female basketball players. Likewise, Zhou et al. [17] reported that 6–8 weeks of plyometric exposure enhanced vertical jump, sprint, and change-of-direction performance in youth athletes. Collectively, these findings suggest that the four-week training period in the present study was likely insufficient for the neutral and low DSI groups to translate their training into measurable gains in explosive or maximal strength performance.

The present findings also suggest that DSI may be a useful tool to guide training prioritization of strength-training emphasis, provided that practitioners interpret the ratio in conjunction with its components (ballistic peak force and maximal isometric peak force). Importantly, testing modality (e.g., IMTP vs. isometric squat) can influence absolute values and athlete classification; therefore, DSI cut-offs should be established and interpreted within the specific testing context.

Although increases in estimated 1RM were observed within some groups, the absence of a significant time × group interaction indicates that these changes should not be interpreted as differential responses between groups.

### Study limitations

These results should nevertheless be interpreted with caution due to several limitations. The absence of a control group prevents definitive attribution of the observed changes solely to the training program. The lack of sprint and change-of-direction assessments limits the ability to evaluate transfer to basketball-specific performance. Finally, heterogeneity in sex, age, and maturational status within the sample may have influenced the magnitude of the responses observed. In addition, external load exposure (e.g., match minutes and individual on-court demands) was not quantified, which may have contributed to inter-individual variability in neuromuscular adaptations. Stratification was based on baseline DSI tertiles for applied decision-making; therefore, group comparisons should be interpreted cautiously due to potential regression-to-the-mean effects. External training load, although standardized in collaboration with coaching staff, was not directly quantified, which may represent a potential confounding factor. Biological maturation status was not assessed, which may influence training responsiveness in youth athletes. The relatively small sample size and subgroup division may limit statistical power.

Future studies should examine longer-duration interventions to determine whether ballistic training can elicit significant improvements in explosive performance and to compare the efficacy of maximal, ballistic, and combined approaches over extended periods. It would also be valuable to stratify participants by sex when establishing DSI cut-off groups, given the known differences in force production between males and females that may influence both training responses and neuromuscular profile classification. Future research should also incorporate sport-specific performance measures such as sprinting and change-of-direction ability to improve ecological validity.

## Conclusions

This study examined neuromuscular changes during a four-week individualized in-season strength-training program based on the Dynamic Strength Index (DSI) in youth basketball players. Athletes classified within the high DSI group exhibited increases in maximal isometric force and estimated 1RM back squat, accompanied by a reduction in DSI values, whereas CMJ performance remained largely unchanged. Given the applied, non-controlled design, these findings should be interpreted as monitoring outcomes rather than definitive causal effects of the training intervention.

From a practical perspective, these observational findings suggest that DSI profiling may assist practitioners in prioritizing training emphasis during the competitive season. DSI values should be interpreted alongside their constituent variables (CMJ peak force and maximal isometric peak force) and within the specific testing context, as these factors influence classification and practical interpretation.

Overall, these findings highlight the importance of maximal strength development within youth athletic populations, although longer intervention periods may be required to translate these changes into improvements in explosive performance.
