# Correction: Neuromuscular changes during a four-week individualized in-season strength-training program based on the dynamic strength index in youth basketball players: A quasi-experimental study

**DOI:** 10.1371/journal.pone.0356736

**Published:** 2026-08-20

**Authors:** Casado-Martínez P, Romero-Moraleda B, González-García J

An additional affiliation is missing for the second author. Romero-Moraleda B is also affiliated with the Department of Physical Education, Sport and Human Movement, Universidad Autónoma de Madrid, Madrid, Spain.
